# Curcumin Suppresses the Colon Cancer Proliferation by Inhibiting Wnt/β-Catenin Pathways via miR-130a

**DOI:** 10.3389/fphar.2017.00877

**Published:** 2017-11-24

**Authors:** Huiqiang Dou, Renhui Shen, Jianxin Tao, Longchang Huang, Haoze Shi, Hang Chen, Yixin Wang, Tong Wang

**Affiliations:** Department of Endoscopy Surgery, Wuxi People’s Hospital Affiliated to Nanjing Medical University, Wuxi, China

**Keywords:** colon cancer, curcumin, Wnt signaling, anti-tumor, miRNA

## Abstract

Curcumin exhibits anti-tumor effects in several cancers, including colorectal carcinoma (CRC), but the detailed mechanisms are still unclear. Here we studied the mechanisms underlying the anti-tumor effect of curcumin in colon cancer cells. SW480 cells were injected into mice to establish the xenograft tumor model, followed by evaluation of survival rate with the treatment of curcumin. The expression levels of β-catenin, Axin and TCF4 were measured in the SW480 cells in the absence or presence of curcumin. Moreover, miRNAs related to the curcumin treatment were also detected *in vitro*. Curcumin could suppress the growth of colon cancer cells in the mouse model. This anti-tumor activity of curcumin was exerted by inhibiting cell proliferation rather than promoting cell apoptosis. Further study suggested that curcumin inhibited cell proliferation by suppressing the Wnt/β-catenin pathway. MiR-130a was down-regulated by curcumin treatment, and overexpressing miR-130a could abolish the anti-tumor activity of curcumin. Our study confirms that curcumin is able to inhibit colon cancer by suppressing the Wnt/β-catenin pathways via miR-130a. MiR-130a may serve as a new target of curcumin for CRC treatment.

## Introduction

Colorectal carcinoma (CRC) is the second most common malignancy in China (Li and Ma, 2014). Advanced high-grade CRC has a five-year survival rate of less than 10% (Jeong and Cairns, 2013; Wang et al., 2013). According to the Global Cancer Statistics, the incidence rate of CRC is rising in East Asia (Center et al., 2009). Current treatments of CRC include surgery and chemotherapy (Mishra et al., 2013). Chemotherapeutic agents such as capecitabine and oxaliplatin are categorized as “special drugs” for the treatment of stage III/IV CRC (Chiu et al., 2014). However, the efficacy of current treatments are compromised by the high recurrence rate and poor prognosis (Fleming et al., 2012). Thus, it is in urgent need to find new therapeutic strategies for CRC treatment.

The Wnt pathway is categorized as canonical and non-canonical signaling pathways, the latter of which is β-catenin independent (Bahrami et al., 2017). On the other hand, for the canonical pathway, in the absence of Wnt ligands, free cytoplasmic β-catenin is destabilized and degraded by a destruction complex composed by APC, Axin, casein kinase 1a (CK1a), and glycogen synthase kinase-3b (GSK3b). Interaction of Wnt with Frizzled and indicates the activation of Wnt signaling. When Disheveled (Dvl) and Axin are recruited to the cell membrane and GSK3b is suppressed, β-catenin is released from of the destruction complex and accumulates in the cytoplasm. Accumulated cytoplasmic β-catenin can be translocated into the nucleus where it binds to various transcription factors, such as T-cell factor (TCF) and lymphoid enhancer factor1 (LEF-1), to activate Wnt target genes (Barker, 2008; Zhan et al., 2017). In most CRC patients, there is at the least one mutation in the Wnt signaling cascade genes, such as the β-catenin and APC (Bahrami et al., 2017). About 80% patients with sporadic colorectal cancers carry APC mutation, while half of colon cancer patients with wild-type APC carry mutations in β-catenin (Morin et al., 1997). In summary, the Wnt/β-catenin pathway becomes the therapeutic target for treating CRC (Xiao et al., 2003).

Curcumin is the major yellow pigment and spice in turmeric and curry, and is a powerful anti-cancer agent (Zhou et al., 2017). Studies indicate that curcumin has anti-tumor effects on several cancers, including CRC (Tong et al., 2016; Guo et al., 2017; Simental-Mendia et al., 2017; Xu et al., 2017), but the detailed mechanisms are still unclear.

In this study, we found that curcumin could suppress colon cancer both *in vitro* and *in vivo*, supporting curcumin as a chemotherapy candidate for colon cancer. The anti-tumor activity of curcumin was demonstrated by repressing the Wnt/β-catenin pathway and inhibiting the proliferation of colon cancer cells. This process was regulated by repressing the expression of microRNA (miR)-130a, and overexpressing miR-130a could completely abolish the curcumin-induced anti-tumor activity in colon cancer.

## Materials and Methods

### Reagents

Dulbecco’s Modified Eagle Medium (DMEM) was bought from GIBCO (Pleasanton, CA, United States), and the fetal calf serum (FCS) was purchased from HyClone Laboratories (Logan, UT, United States). Anti-human β-catenin, Axin, TCF4, GAPDH antibodies and curcumin were purchased from Sigma–Aldrich (St. Louis, MO, United States). Cell counting kit-8 was purchased from Dojindo Laboratories (Tokyo, Japan).

### RNA Extraction and q-PCR

Total RNA was isolated with a mirVana^TM^ miRNA Isolation Kit (Ambion, Austin, TX, United States). All primers for detecting miRNA expression were designed and synthesized by Genscript Co. Ltd. (Nanjing, China) using the mirVana^TM^ qRT-PCR Primer Sets. U6 was used as internal control to normalize the expression levels of each miRNA. The fold change in miRNA expression was determined by comparative CT method.

### Cell Culture

The colon cancer cell line SW480 was obtained from American Type Culture Collection (Manassas, VA, United States) and cultured in DMEM supplemented with 10% FCS and 1% penicillin/streptomycin (Sigma–Aldrich).

### Flow Cytometry and Apoptosis

Cells with different treatments were washed twice in FACS medium (phosphate buffered saline containing 1% FCS and 0.1% NaN_3_). Then the cells were washed three times with Annexin V binding buffer. After centrifugation, the supernatant was discarded. Cells were incubated for 30 min at 4°C with FITC-Annexin V according to standard procedure. PI was added before testing. Fluorescence was measured using a FACSCalibur (Becton Dickinson, San Diego, CA, United States), and data were analyzed by the Flowjo Software (Becton Dickinson, San Diego, CA, United States).

### Western Blot Analysis

1 × 10^7^ cells were lysed in a buffer containing 20 mM Tris-HCl (pH 7.6), 250 mM NaCl, 0.5% NP-40, 3 mM EDTA, and 1.5 mM EGTA with 10 μg/ml Aprotinin, 10 μg/ml leupeptin, 1 mM DTT, 1 mM PNPP and 0.1 mM Na_3_VO_4_ as protease and phosphatase inhibitor. After centrifugation, cell lysates (100 μg/lane) were subjected to 10% SDS-PAGE and transferred onto polyvinylidene difluoride membranes (Roche, Germany). The membranes were blocked for 1 h in TBST (25 mM Tris-HCl, pH 7.6, 125 mM NaCl, 0.1% Tween-20) containing 5% nonfat dried milk, and then incubated with appropriate antibodies diluted in TBST containing 5% nonfat dried milk at 4°C overnight. HRP-conjugated secondary antibodies were from Sigma. Protein bands were detected by the Immobilon Western Chemiluminescent HRP Substrate (Millipore, United States) and images were taken by FluorChem FC2 System (Alpha Innotech Corporation, United States).

### Cell Proliferation Assay

The cell proliferation was detected by cell counting or by Cell Counting Kit (CCK)-8 according to the manufacturer’s instructions.

### BrdU Incorporation Assay

The 5-bromo-2′-deoxyuridine (BrdU) (B5002, Sigma) was incorporated into cellular DNA during cell proliferation. PE-conjugated BrdU mouse monoclonal antibody (#50230, Cell signaling Technology) was used to label the BrdU. After removing labeling medium, cells were fixed and DNA was denatured with fixing/denaturing solution. FACS was used to detect the incorporated BrdU by the PE channel.

### Animals and Surgical Procedures

6-week-old female nude mice were provided by Animal Center in Nanjing Medical University. All protocols, described below, were approved by the Animal Care and Use Committee at Wuxi People’s Hospital Affiliated to Nanjing Medical University. 1 × 10^5^ SW480 cells suspended in 1 ml DMEM were injected by axillary inoculation. Curcumin was injected by intraperitoneal (i.p.) injection at the dose of 200 mg/kg for 5 days after tumor generation. Mice were monitored daily to record the survival rate.

### Statistical Analysis

Results were expressed as mean ± SD. The one-way analysis of variance (ANOVA) was used to determine significant differences between the means of three or more independent groups, and the Student’s *t*-test was to examine the significance between two groups. *P*-values (*p*) were indicated in each figure.

## Results

### Curcumin Exhibited Anti-tumor Activity

**FIGURE 1:**
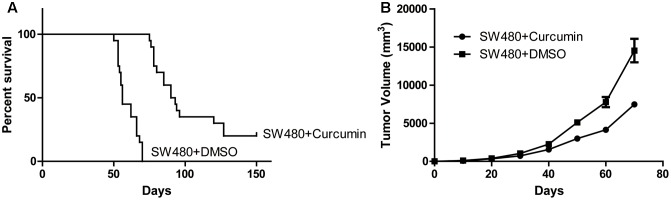
Curcumin suppressed colon cancer. **(A)** Tumor generated by SW480 with different treatment as indicated was measured in diameters and calculated to volume according to the time point. *n* = 20 for each group. **(B)** Survival curve of the mice treated with curcumin or DMSO. Each group contains 20 mice.

Since curcumin was reported to have anti-tumor effects on colon cancer, we first used colon cancer cell line SW480 to generate the *in vivo* animal model by subcutaneous injection. The mice with the tumor receiving intraperitoneal injection of curcumin showed prolonged life span compare to the control group receiving vehicle injection (**Figure 1A**). Meanwhile, the tumors in the curcumin group grew slower than the control group (**Figure 1B**). These data indicated that curcumin exhibited anti-tumor activity in the *in vivo* mouse model generated by the SW480 cell line.

### Curcumin Suppressed Proliferation of Tumor Cells

**FIGURE 2:**
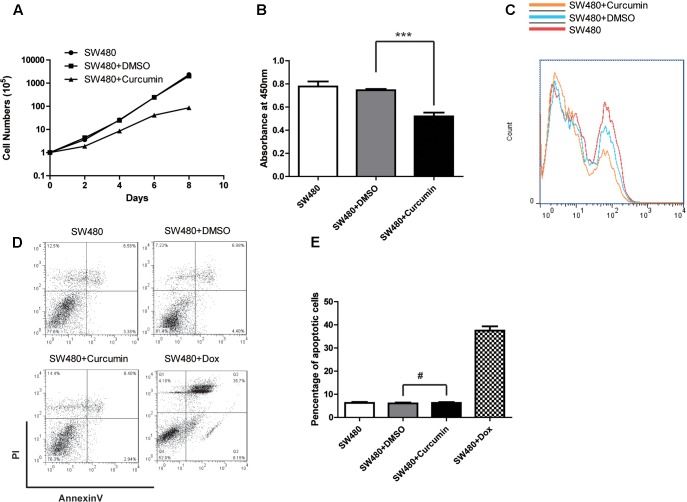
Curcumin inhibited the cell proliferation of SW480. **(A)** Cell proliferation of different groups as indicated was determined by cell counting. The concentration of curcumin was 40 μM. Data were represented as mean ± SD; *n* = 3 independent experiments. **(B)** CCK-8 kit was used to evaluate the viability of each group of the cells as indicated. Data were represented as mean ± SD; *n* = 3 independent experiments. ^∗∗∗^*p* < 0.001. **(C)** Cell proliferation was detected by BrdU incorporation assay and *n* = 3 independent experiments and this panel presented one of these repeats. **(D)** The apoptosis of the SW480 cells with different treatment as indicated was determined by using the Annexin-V-FITC & PI Apoptosis Kit and assessed by flow cytometry. *n* = 3 independent experiments and this panel presented one of these repeats. **(E)** Statistic of percentage of the apoptosis cells performed in **(D)**. Data showed the Annexin-V and PI double positive cells. Data were represented as mean ± SD; *n* = 3 independent experiments. ^#^*p* > 0.05.

In order to investigate the mechanism underlying the inhibitory effect of curcumin on colon cancer cells, we performed assays to evaluate cell proliferation and apoptosis. In the cell counting assay, we found that the cells treated with curcumin grew much slower, compared to both the control group and the group treated with DMSO (**Figure 2A**), which indicated that curcumin inhibited cell proliferation *in vitro*. Consistent with cell counting assay, results from the CCK-8 kit for determining the viability of the colon cancer cells also indicated that the curcumin-treated cells showed the lowest viability (**Figure 2B**). Next, we tested the apoptosis of SW480 cells with different treatments. Percentages of the Annexin V-positive apoptotic cells with curcumin treatment did not display any difference compared to the control cells (**Figures 2C–E**). These results indicated that curcumin suppressed colon cancer cells by inhibiting proliferation rather than promoting apoptosis. It is worth noting that similar results could be observed in another colon cancer cell line HCT116 (Supplementary Figures S1A–C).

### Curcumin Inhibited Wnt/β-Catenin Pathways

**FIGURE 3:**
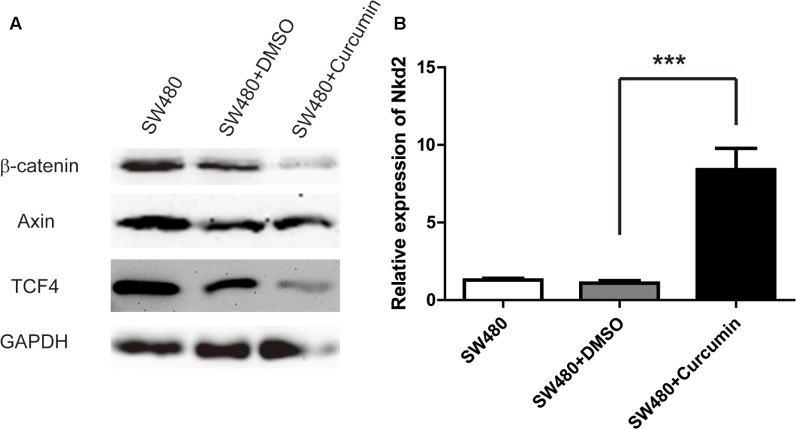
Curcumin inhibited Wnt/β-catenin pathways. **(A)** Western blot analysis of the protein expression of β-catenin, axin, TCF4, and GAPDH in SW480 cells following curcumin treatment for 24 h. The concentrations of curcumin was 40 μM. *n* = 3 independent experiments and this panel presented one of these repeats. **(B)** RT-q-PCR of the mRNA level of Nkd2 in SW480 cells with the treatments as indicated. Data were represented as mean ± SD; *n* = 3 independent experiments. ^∗∗∗^*p* < 0.001.

The Wnt/β-catenin pathways play an important role in colon cancer by promoting cell proliferation. Therefore, we asked whether the anti-tumor activity of curcumin was mediated through Wnt/β-catenin signaling. Western bolt analysis showed that the expression level of β-catenin was decreased after curcumin treatment, whereas the expression level of Axin was unchanged (**Figure 3A**). Since Wnt/β-catenin pathways have many negative regulators, such as Nkd2, we assessed the mRNA level of Nkd2 by RT-qPCR, and found that Nkd2 was upregulated by curcumin treatment (**Figure 3B**). We then proceeded to assess the Wnt target gene TCF4. Although TCF is a transcription factor in the Wnt/β-catenin signaling, it is also a Wnt target gene. Consistent with our observations on Nkd2 and β-catenin, TCF4 was also down-regulated by curcumin treatment. The above results demonstrated that curcumin inhibited colon cancer cell proliferation through suppressing the Wnt/β-catenin signaling pathways.

### Curcumin Suppressed the Wnt/β-Catenin Pathway via miR-130a

**FIGURE 4:**
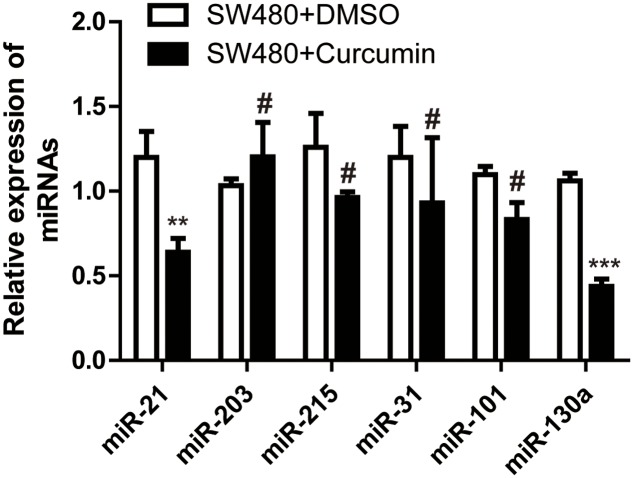
Curcumin regulated several miRNAs in SW480 cells. Q-PCR showing the expression level of miRNAs as indicated. Data were represented as mean ± SD; *n* = 3 independent experiments. ^∗∗^*p* < 0.01, ^∗∗∗^*p* < 0.001, ^#^*p* > 0.05.

**FIGURE 5:**
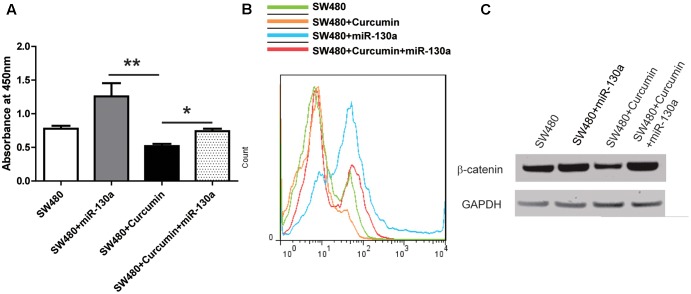
Curcumin inhibited Wnt pathway via miR-130a. **(A)** CCK-8 kit was used to evaluate the viability of each group of the cells as indicated. Data were represented as mean ± SD; *n* = 3 independent experiments. ^∗^*p* < 0.05, ^∗∗^*p* > 0.05. **(B)** Cell proliferation was detected by BrdU incorporation assay and *n* = 3 independent experiments and this panel presented one of these repeats. **(C)** Western blot analysis of the protein expression of β-catenin and GAPDH in SW480 cells. *n* = 3 independent experiments and this panel presented one of these repeats.

In order to reveal the mechanism underlying curcumin regulation on the Wnt/β-catenin signaling, we tested several miRNAs reported to be upregulated in CRC. q-PCR showed that with curcumin treatment, miR-21 and miR-130a were down-regulated (**Figure 4**). MiR-21 has been reported to be involved in colon cancer, whereas the function of miR-130a is not clear. Therefore, we overexpressed miR-130a in SW480 cells and treated the cells with curcumin, followed by determining cell proliferation and β-catenin expression level. Interestingly, in the presence of miR-130a, cell proliferation was restored to similar level as the control group (**Figure 5A**), and β-catenin level was also returned to normal level (**Figure 5B**). These results indicated that curcumin suppressed the Wnt/β-catenin pathway via inhibiting miR-130a.

**FIGURE 6:**
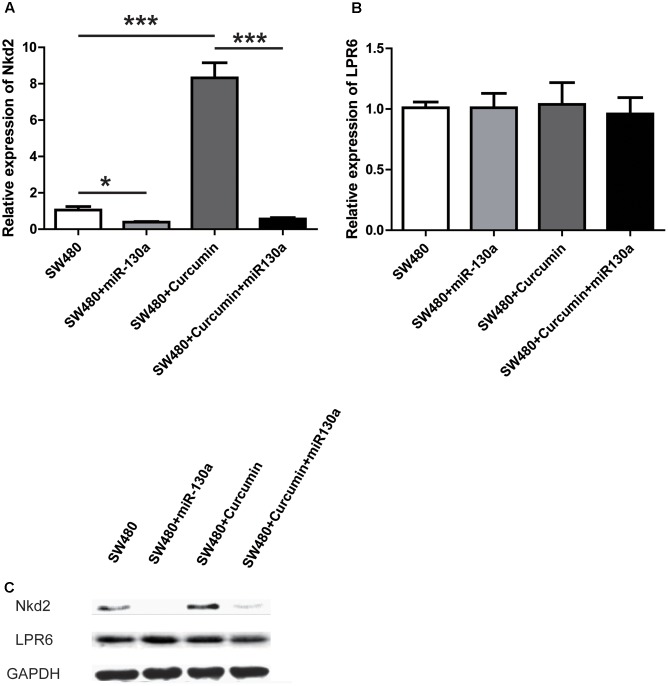
miR-130 abolished the anti-proliferation effect of curcumin by downregulating nkd2. **(A)** RT-q-PCR of the mRNA level of Nkd2 in SW480 cells with the treatments as indicated. Data were represented as mean ± SD; *n* = 3 independent experiments. ^∗^*p* < 0.05, ^∗∗∗^*p* < 0.001. **(B)** RT-q-PCR of the mRNA level of LPR6 in SW480 cells with the treatments as indicated. Data were represented as mean ± SD; *n* = 3 independent experiments. **(C)** Western blot analysis of the protein level of NKD2 and LPR6 in SW480 cells with the treatment as indicated. *n* = 3 independent experiments and this panel presented one of these repeats.

Furthermore, we investigated potential mechanism underlying miR-130a involvement in Wnt signaling. We first evaluated Nkd2 expression, since it has been shown to be up-regulated by curcumin treatment (**Figure 3**). Our results showed that miR-130a could repress the expression of Nkd2 in both mRNA and protein levels (**Figures 6A,C**). We also predicted the targets of miR-130a through online tool^1^, among which LPR6 was identified to be related to the Wnt pathway. Therefore, we tested the level of LPR6 after miR-130a overexpression in the absence or presence of curcumin treatment. Unfortunately, neither the mRNA nor the protein level of LPR6 was changed (**Figures 6B,C**). We observed similar pattern in these proteins in another colon cancer cell line HCT116 (Supplementary Figure S1D). However, none of the predicted target genes of miR-130a were found to be potential indirect regulators of Nkd2 (data not shown), and 5′-UTR of Nkd2 was also not found to be targeted by miR-130a using TargetscanHuman 7.1^2^.

**FIGURE 7:**
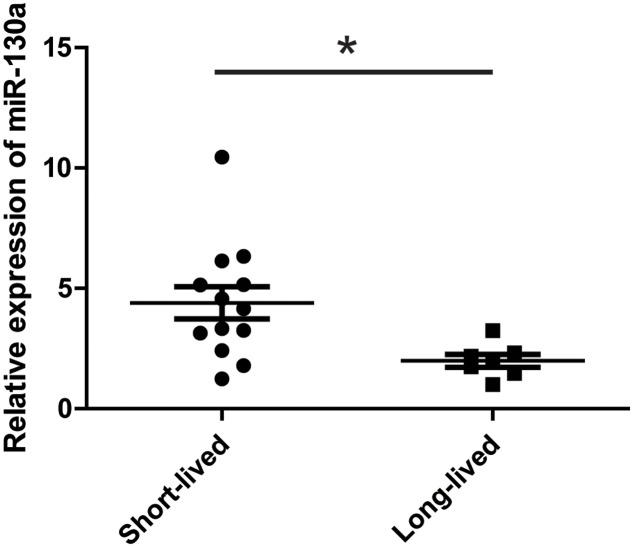
miR-130a showed higher level in the short-lived mice treated with curcumin. Mice treated with curcumin in **Figure 1A** (*n* = 20) was divided into two groups. Short-lived group indicated the mice lived less than 100 days (*n* = 13) while long-lived group indicated the mice lived more than 100 days (*n* = 7). Q-PCR showed the expression level of miRNAs in the serum of each mice. ^∗^*p* < 0.05.

These data suggested that miR-130 might act to antagonize curcumin’s anti-tumor effect. We therefore examined the serum level of miR-130a in mice treated with curcumin. We divided the mice into two sub-groups: short-lived group (<100 days, 13 mice) and long-lived group (>100 days, 7 mice, 4 of which survived more than 150 days). The serum level of miR-130a was significantly higher in the short-lived group than that of the long-lived group of mice (**Figure 7**), which confirmed that miR-130a functioned to antagonize curcumin’s anti-tumor effect.

## Discussion

Curcumin, the major curcuminoid and the most active component in turmeric, is a biologically active phytochemical (Goel et al., 2008). Curcumin is also reported to be beneficial in arthritis (Ahmed et al., 2005; Chandran and Goel, 2012), neurodegenerative diseases (Ramassamy, 2006; Cole et al., 2007) and autoimmune conditions (Bright, 2007). Increasing evidences have shown that curcumin exhibits anti-tumor activity (Aggarwal et al., 2003; Kunnumakkara et al., 2008; Kasi et al., 2016) in several cancers, including CRC (Tong et al., 2016; Guo et al., 2017; Simental-Mendia et al., 2017; Xu et al., 2017), but the detailed mechanism is still unclear.

In this study, we first confirmed the anti-tumor activity of curcumin in colon cancer *in vivo* using a mouse model. Curcumin exerts its anti-tumor activity by inhibiting proliferation rather than promoting apoptosis of the colon cancer cells. Then we attempted to identify the signaling pathway involved in this process. In particular, curcumin affects several signaling pathways known to be important for solid and blood cancers, such as the PI3k/Akt-1/mTOR pathway (Sokolosky et al., 2011; McCubrey et al., 2012; Zhao et al., 2016; Zhu et al., 2017), the Ras/Raf/MEK/ERK pathway (McCubrey et al., 2012), the GSK-3beta pathway (Yun et al., 2015), p53 activity (Sidhar and Giri, 2017), and NF-κB pathways (Marin et al., 2007). In addition, the Wnt signaling pathway is one of the key dysregulated pathways in colorectal cancer, which increases cell proliferation and resistance to chemotherapy (Park et al., 2012; Moradi et al., 2017). Therefore we tested Wnt signaling in the colon cancer cells treated with curcumin, and our results showed that curcumin could inhibit the Wnt signaling by down-regulating β-catenin, which was mediated by up-regulating the Wnt signaling negative regulator Nkd2. These results demonstrated that curcumin exerted anti-tumor activity by inhibiting Wnt signaling to inhibit the cell proliferation.

Next, we investigated the mechanism underlying curcumin effect on Wnt signaling. In addition to the gene expression studies (Ramachandran et al., 2005; Teiten et al., 2009; Huminiecki et al., 2017), we noticed some microRNA (miRNA) studies (Ye et al., 2015; Zhou et al., 2017) implicating miRNAs in the function of curcumin. MiRNAs are short non-coding RNAs of 20–24 nucleotides that play important roles in many biological pathways including processes related to cancer, such as proliferation, cell cycle control, apoptosis, differentiation, migration and metabolism. We therefore examined the miRNAs reported to be involved in colon cancer, and found that only miR-21 and miR-130a were affected by curcumin. Since miR-130a was a novel miRNA that had few reported functions in colon cancer, we focused on the role of miR-130a in curcumin action in colon cancer. We overexpressed miR-130a in SW480 cells and treated them with curcumin. The results showed that curcumin could suppress the proliferation of SW480 cells. In the presence of miR-130a, cell proliferation was restored to similar level as the control cells. We also assessed the Wnt/β-catenin signaling in the colon cancer cells. Consistent with the proliferation results, the protein level of β-catenin was also restored to normal level.

Subsequently, we found that miR-130a could repress the expression level of Nkd2, which could be up-regulated by curcumin. These results suggested that miR-130a may regulate the Wnt pathway by inhibiting Nkd2. However, none of the predicted target genes of miR-130a were found to be potential indirect regulators of Nkd2, therefore the exact mechanism under this inhibition still elusive, and further investigations are warranted. It has been reported that miR-130a expression is disregulated in several types of cancer (Zhang et al., 2017), including colon cancer (Kara et al., 2015). Increasing targets of miR-130a have been identified. For example, TNF-α can activate NF-κB activity to upregulate miR-130a, which in turn targets and inhibits TNF-α expression as a negative feedback loop. This negative feedback regulation of NF-κB/miR-130a/TNF-α/NF-κB may provide insight into the carcinogenesis of cervical cancer (Zhang et al., 2014). MiR-130a also increases drug resistance by regulating RUNX3 and Wnt signaling in cisplatin-treated hepatocellular carcinoma cells (Xu et al., 2012). However, little is known about the targets of miR-130a in colon cancer. Here our results proposed that Wnt pathway might be a potential target for miR-130a in colon cancer, but its direct target still needs to be identified in further work.

Furthermore, we have verified that curcumin exhibits anti-tumor activity in colon cancer, which was exerted by inhibiting the Wnt/β-catenin signaling pathway to suppress cell proliferation in the colon cancer cells. Our results further suggested that curcumin could affect levels of several miRNAs in colon cancer cells. Among these miRNAs, miR-130a may be important for cancer formation because overexpressing miR-130a could restore the proliferation of curcumin-suppressed cancer cells back to normal. Meanwhile, the expression of Wnt signaling pathway component, β-catenin, was also restored to similar level as the original cancer cells. These results indicated that curcumin inhibited the Wnt signaling by repressing the expression level of the miR-130a. Furthermore, we tested the serum level of miR-130a in the experimental mice. We found that the short-lived mice had higher level of miR-130a in their serum than the long-lived mice. This suggested that miR-130a might contribute to the resistance of colon cancer cells to chemotherapy. Our study supports miR-130a as a novel target in the treatment of colon cancer. Taken together our earlier clinical study where high miR-130a expression level was significantly associated with poor clinical outcome (Kara et al., 2015), results in our current study suggests that when agent like curcumin is used to treat colon cancer, therapies inhibiting miR-130a may be combined for better clinical efficacy.

## Conclusion

Curcumin suppresses colon cancer by inhibiting Wnt/β-catenin pathway via miR-130a. MiR-130a may serve as a new target for CRC treatment.

## Author Contributions

HD, RS, JT, LH, HS, HC, and YW performed the experiments and analyzed the data. TW wrote the manuscript and designed the study.

## Conflict of Interest Statement

The authors declare that the research was conducted in the absence of any commercial or financial relationships that could be construed as a potential conflict of interest.

## Footnotes

1 http://www.microrna.org

2 http://www.targetscan.org/vert_71/
