# Supplementary material for: Curcumin Suppresses the Colon Cancer Proliferation by Inhibiting Wnt/β-Catenin Pathways via miR-130a
Source: Front Pharmacol. 2017 Nov 24;8:877. doi: 10.3389/fphar.2017.00877 (PMC5705620; doi:10.3389/fphar.2017.00877)
Supplement: Supplementary file 1 [file Presentation_1.PDF]

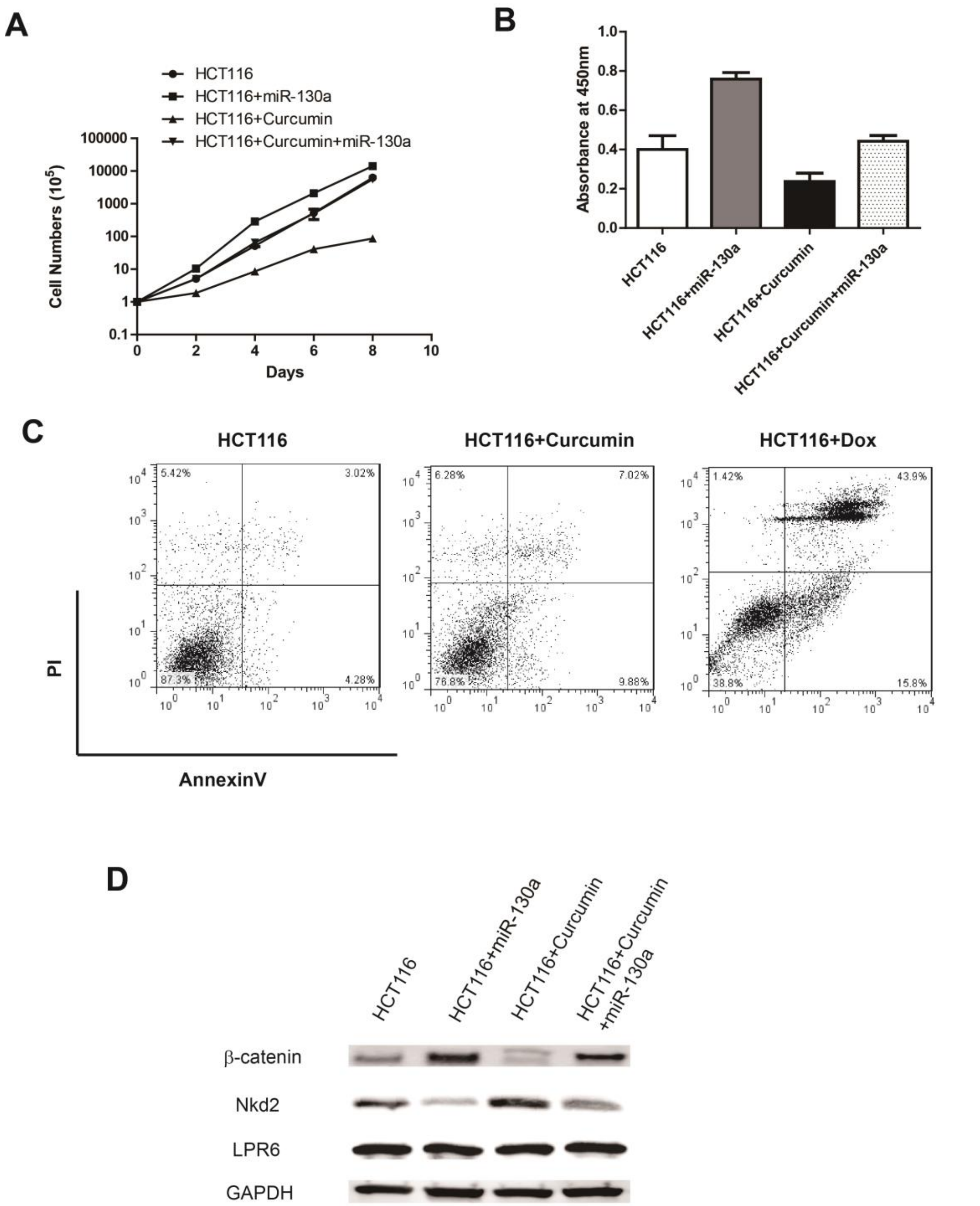

Supplementary Figure S1. **Curcumin inhibited the cell proliferation of HCT116 by inhibiting miR-130a.**

(A) Cell proliferation of different groups as indicated was determined by cell counting. Data were represented as mean ± s.d.; n = 3 independent experiments.

(B) Cck-8 kit was used to evaluate the viability of each group of the cells as indicated. Data were represented as mean ± s.d.; n = 3 independent experiments.

(C) The apoptosis of the HCT116 cells with different treatment as indicated was determined by using the Annexin-V-FITC & PI Apoptosis Kit and assessed by flow cytometry. n = 3 independent experiments and this panel presented one of these repeats.

(D) Western blot analysis of the protein level of β-catenin, NKD2 and LPR6 in HCT116 cells with the treatment as indicated. n = 3 independent experiments and this panel presented one of these repeats.
